# RNA-seq co-expression network analysis reveals anxiolytic behavior of mice with Efnb2 knockout in parvalbumin+ neurons

**DOI:** 10.1186/s13041-021-00829-z

**Published:** 2021-07-19

**Authors:** Ying Sun, Le Ma, Jianhua Chen, Weidi Wang, Shiyu Peng, Ying Cheng, Yu Zhang, Jinghong Chen, Peijun Ju

**Affiliations:** 1 Shanghai Mental Health Center, Shanghai Jiao Tong University School of Medicine, Shanghai Key Laboratory of Psychotic Disorders, Shanghai, 200240 China; 2 King’s Lab, Shanghai Jiao Tong University School of Pharmacy, Shanghai, 200240 China; 3 School of Life Sciences, Westlake Institute for Advanced Study, Westlake University, Hangzhou, 310000 China

**Keywords:** Efnb2, Anxiety, RNA-seq, WGCNA

## Abstract

Anxiety disorders are the most common psychiatric disorders, and the change in the activity of the prefrontal cortex (PFC) is considered as the underlying pathological mechanism. Parvalbumin-expressing (PV+) inhibition contributes to the overall activity of the PFC. However, the molecular mechanism underlying the excitation-inhibition imbalance of PV+ neurons in the PFC is unknown. Efnb2 is a membrane-bound molecule that plays an important role in the nervous system through binding the Eph receptor. To investigate whether the loss of Efnb2 in PV+ affects anxiety, we examined the behavior of wild type and Efnb2 in PV+ neurons knockout (KO) mice. We monitored the defensive responses to aversive stimuli of elevated plus maze (EPM) and found that KO mice exhibited obvious fearless and anxiolytic behaviors. To further investigate the underlying regulatory mechanism, we performed RNA sequencing, analyzed the differentially expressed genes (DEGs), and constructed the weighted gene co-expression network analysis (WGCNA). The WGCNA identified 12 characteristic modules. Among them, the MEgreen module showed the most significant correlation with KO mice of EPM stimuli. The Gene Ontology enrichment and the Kyoto Encyclopedia of Genes and Genomes enrichment analysis revealed that this was related to the distal axon, Ras signaling pathway and insulin signaling pathway. Furthermore, the whole-cell voltage clamp recordings also proved that Efnb2 gene knock-out could affect synaptic function. Together with the transcriptomic analysis of mice with Efnb2 knockout on PV+ neurons, our findings suggest that Efnb2 gene in the PV+ neuron of PFC may be a crucial factor for fear and anxiety, which provide an insight into anxiety pathophysiology.

**Supplementary Information:**

The online version contains supplementary material available at 10.1186/s13041-021-00829-z.

## Introduction

Anxiety is one of the most common mental disorders that can produce adverse cognitive effects and contribute to the clinical manifestation of anxiety disorders. Anxiety disorders are of considerable morbidity [1]. They are commonly comorbid with other mental disorders like major depression, dysthymia, and so on. These disorders will undoubtedly increase the rates of disability and the risk of mortality. The balance of excitation and inhibition in prefrontal cortex (PFC) plays a fundamental role in the emotion regulation of anxiety [2]. The excitation and inhibition balance is regulated by the interaction of glutamatergic and GABAergic neurons. Specifically, the prefrontal GABAergic interneurons, especially the Parvalbumin-expressing (PV+) neurons, are the primary regulators of the projective excitatory neurons’ spiking activity and emotional behavior [3]. The PV+ neurons account for more than 50% of the total population of GABAergic neurons in the frontal cortex and control the region near or on the cell bodies of excitatory pyramidal cells by providing strong, fast spiking inhibition [4, 5]. Therefore, understanding the underlying mechanism remains a priority to promote the development of efficacious and reliable pharmacological anxiolytic therapies.

Ephrins are membrane-bound molecules that play an important role in the nervous system through the binding of the Eph receptor [6, 7]. Ephrin-Eph signaling is involved in mediating the communication among neurons and between neurons and glial cells [8]. For example, the Ephrin B2 (Efnb2) extracellular portion associates with NMDA neurotransmitter receptors and promotes their clustering at synapses following ephrin-B stimulation [9]. Efnb2, a more powerful player compared with ephrin b1 and b3, was found to be expressed in a large proportion of forebrain inhibitory neurons [10]. It has recently been implicated in GABAergic circuit development and considered as a potent regulator of neuronal development and synaptic function [11–14]. Important advances in the studies of Efnb2 in the developing nervous system included axon and dendrite growth [11, 12], dendritic spine morphogenesis, and synapse formation [13, 14]. Reverse signaling by postsynaptic Efnb2 plays an essential role in synaptic plasticity in mutant mice [8, 15]. However, the underlying mechanisms of the pathogenic variation of the Efnb2 in the anxiolytic effect is currently unclear. Combining the existing researches of PV+ neurons and Efnb2 [2, 9], we speculated that the Efnb2-mediated dysfunctions of specific PV+ neurons lead to the imbalanced neural networks.

Efnb2 is an important factor in the neurological system, but the association between Efnb2 gene and anxious stress remains controversial. In this paper, we knocked out the Efnb2 gene of PV+ interneurons in the mouse PFC brain region. To investigate whether genetic Efnb2 variants affect fear and anxiety behaviors, we tested the behavior of mice using the experiments of marble burying, cold stress defecation, and elevated plus maze (EPM) between wild-type (WT) and knock-out (KO) mice. The EPM is an animal model of fear and anxiety and is related to physiology, pharmacology, and behavior [16, 17]. Moreover, EPM has been used as a stress model to investigate the regulatory mechanism of anxiety [18]. Placing an animal on an elevated platform, especially the open arms of the maze, could induce stress in animals and hence increase fear and anxiety.

In this study, we deleted Efnb2 specifically in PV+ neurons and observed fearless and anxiolytic behaviors in KO mice. Furthermore, by performing RNA sequencing, weighted gene co-expression network analysis (WGCNA) and whole-cell patch-clamp recording, we aimed to determine gene co-expression networks underlying fearless and anxiolytic behaviors and identify key modules associating with it, which may provide a treatment strategy and therapeutic target to regulate fear and anxiety symptoms.

## Results

### Mice with Efnb2 knockout on PV+ neurons show obvious anxiolytic behavior

**Fig. 1:**
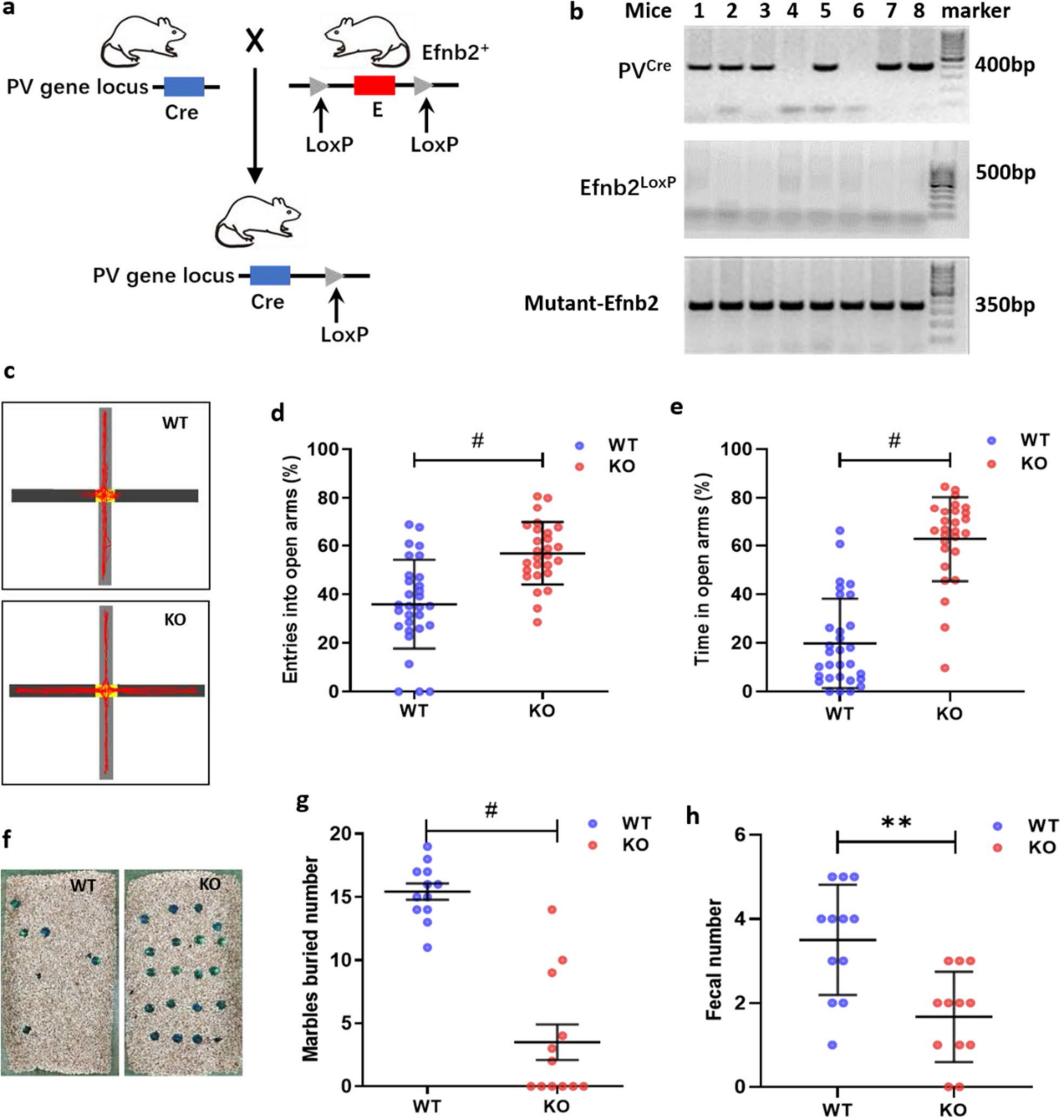
Behavioral results of wild-type (WT) and Efnb2 on PV+ neurons knock-out (KO) mice. **a** Schematic drawing of the breeding strategy for a generation of KO mice. **b** PCR-based analysis of mice genotypes. **c** Mouse movement track diagram. Black: open arms; grey: closed arms; red: tracks. **d** Results of entries into open arms (male, n = 30, 27; Mann Whitney test, ^#^*P* ≤ 0.0001). **e** Results of time in open arms (male, n = 30, 27; Mann Whitney test, ^#^*P* ≤ 0.0001). **f** marbles buried diagram. **g** marbles buried number (male, n = 12, 12; Mann Whitney test, ^#^*P* ≤ 0.0001). **h** Fecal number (male, n = 12, 12; Mann Whitney test, **P ≤ 0.01)

We generated mice lacking Efnb2 only in PV+ neurons by crossing mice with Cre driven by the PV and mice carrying Efnb2 flanked LoxP sites (Fig. 1a). Cre-recombination and mouse genotypes were confirmed by RT-PCR analysis. The Efnb2^LoxP^ primers resulted in gene products of 500 bp. The PV^cre^ primers resulted in gene products of 400 bp. The Mutant-Efnb2 primers resulted in gene products of 350 bp. The mice with loxP-flanked alleles in Efnb2 were WT group, the mice with Efnb2 gene knockout were KO group (mice 1–3, 5, 7, 8; Fig. 1b).

To investigate whether ablation of Efnb2 gene on PV+ neurons was required for the anxiolytic effect elicited on PFC, we performed a behavioral examination through a series of testing trials with marble burying, cold stress defecation, and EPM between WT and KO mice. We chose these behavioral paradigms, because we were mostly interested in determining the innate anxiolytic role of Efnb2. Mice are free to avoid the aversive stimulus or approach the potential threat. These behavioral tests all showed that KO mice exhibited obvious fearless and anxiolytic behaviors. In the EPM experiments, both entries into open arms (%) and time spent in open arms (%) of KO mice were significantly higher than that of WT mice (Mann Whitney test, ^#^P ≤ 0.0001; Fig. 1c, d, and e). In the marble burying and cold stress defecation experiments, KO mice had lower number of marbles buried (Mann Whitney test, ^#^P ≤ 0.0001; Fig. 1f and g) and fecal number (Mann Whitney test, **P ≤ 0.01; Fig. 1h) than the WT group.

### Differential expression genes among WT, KO, WT_EPM and KO_EPM

**Fig. 2:**
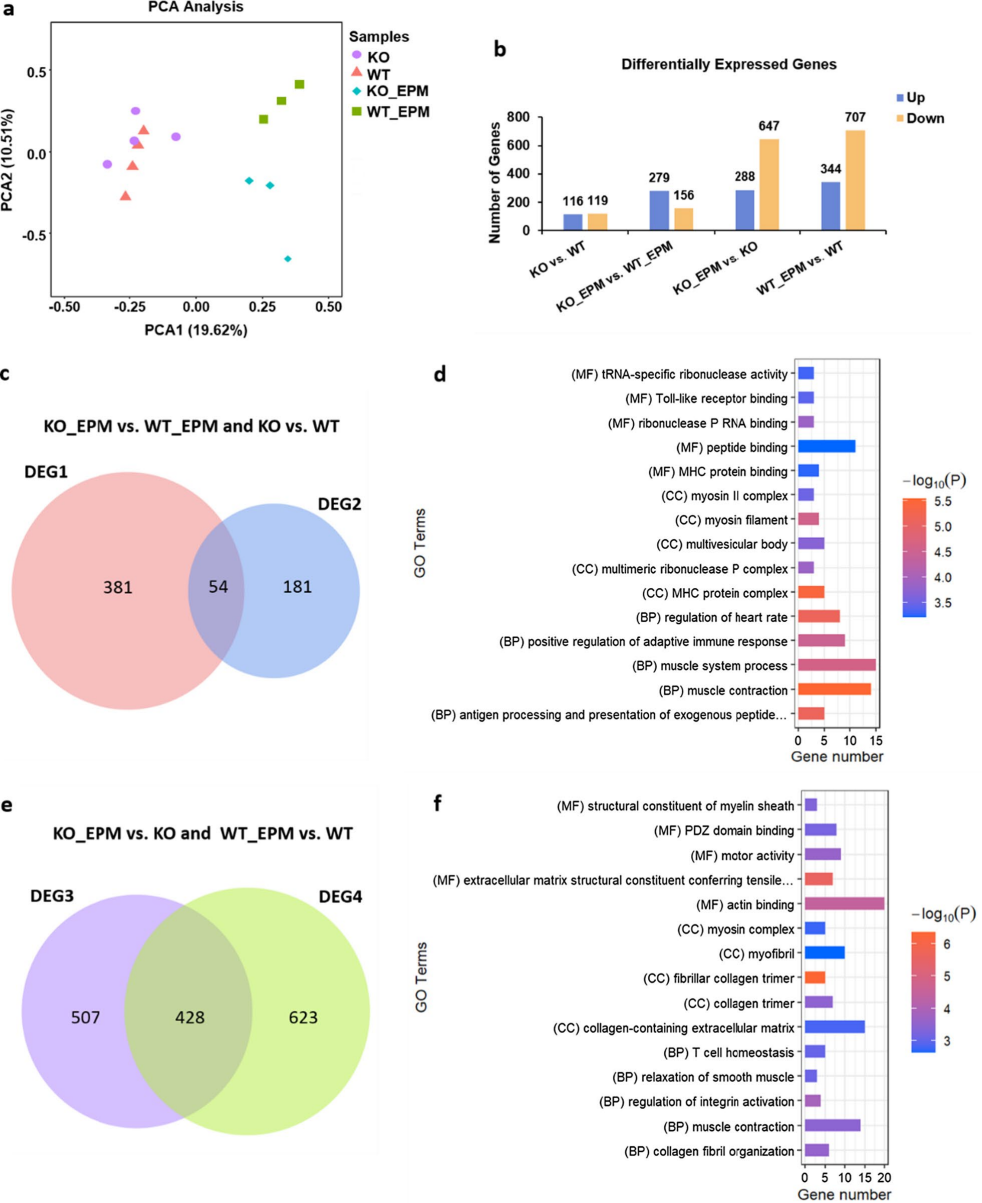
Analysis of differentially expressed genes (DEGs). **a** Principal component analysis (PCA) of WT, KO, WT_EPM, and KO_EPM. **b** The numbers of up-regulated (blue) and down-regulated (yellow) DEGs between KO vs. WT, KO_EPM vs. WT_EPM, KO_EPM vs. KO and WT_EPM vs. WT. **c** Venn diagram of DEGs (KO vs. WT) and DEGs (KO_EPM vs. WT_EPM). **d** The top 5 GO terms of 249 genes (DEG1), which were unshared DEGs in KO_EPM vs. WT_EPM. **e** Venn diagram of DEGs (KO_EPM vs. KO) and DEGs (WT_EPM vs. WT). **f** The top 5 GO terms of 507 genes (DEG3), which were unshared DEGs in KO_EPM vs. KO. GO terms are sorted by the P value. Gene number represents the number of gene types. *BP* biological process, *CC* cellular component, *MF* molecular function

To further explore the molecular determinants of the neural network and behavioral abnormalities in Efnb2 knockout mice, RNA sequencing was performed on the mPFC of the WT (male; n = 4), KO (male; n = 4), WT mice after 1 h of the EPM test (WT_EPM; male; n = 3), and KO mice after 1 h of the EPM test (KO_EPM; male; n = 3). Among the samples, 32.35–53.19 million raw reads were generated. After removing low-quality reads, 31.60–50.92 million clean reads were obtained. Approximately 97.0% of clean reads per sample were successfully mapped to the mouse reference genome (Additional file 1). Finally, 55385 transcripts were obtained and the Fragments Per Kilobase of transcript per Million mapped reads (FPKM) were calculated. The principal component analysis (PCA) found a clear separation between the KO_EPM and WT_EPM, and an overlap was found between the KO and WT (Fig. 2a). We also analyzed the differentially expressed genes (DEGs; Additional file 2) between each group with Deseq2 package in R. Based on the statistical analysis (|Fold change|> 2, Padj < 0.05), 235 (KO vs. WT) genes were dysregulated, of which 116 were up-regulated in the KO group of mice. In other comparisons, 435 (KO_EPM vs. WT_EPM), 935 (KO_EPM vs. KO) and 1051 (WT_EPM vs. WT) genes were dysregulated. We found that the number of dysregulated genes induced by EPM was higher than of dysregulated genes induced by genotype (Fig. 2b).

The Venn diagram of DEGs (KO vs. WT) and DEGs (KO_EPM vs. WT_EPM) showed that there were 381 genes, which were unshared DEGs in KO_EPM vs. WT_EPM (DEG1, Fig. 2c). GO enrichment analysis of the 381 genes showed that they were enriched in the ribonuclease P RNA binding, Toll-like receptor binding, MHC protein binding, peptide binding, and others (Fig. 2d). There were 507 genes, which were unshared DEGs in KO_EPM vs. KO (DEG3, Fig. 2e). The GO enrichment results of the 507 genes revealed the actin binding, structural constituent of myelin sheath, PDZ domain binding, and others (Fig. 2f; Additional File 3).

### WGCNA identify the co-expression module

**Fig. 3:**
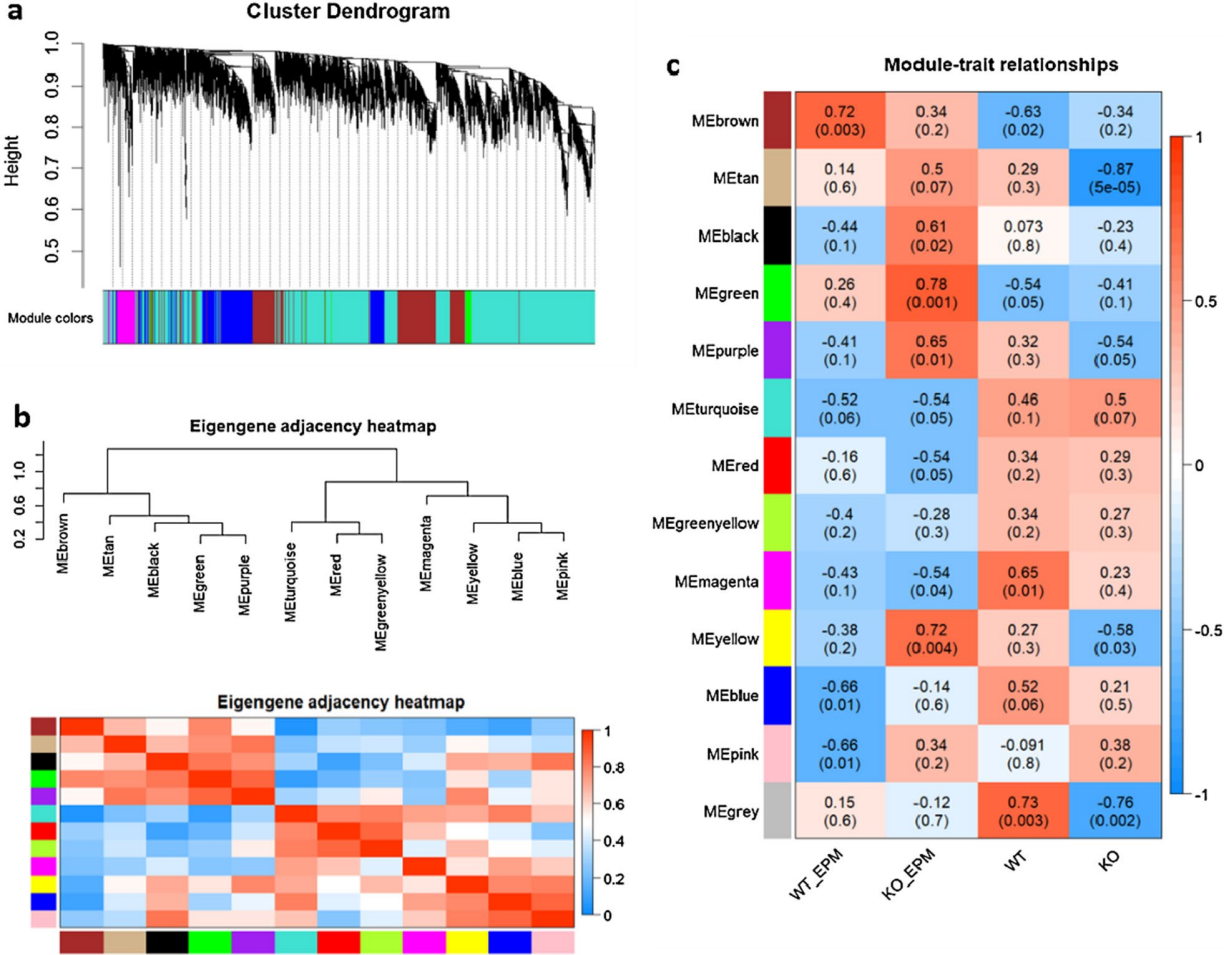
Weighted gene co-expression network analysis (WGCNA). **a** Cluster dendrogram of co-expression networks. **b** Heatmap of WGCNA module-to-module correlation. **c** Heatmap of the correlation between WGCNA module eigengene and sample traits. Within each table cell, upper values indicate the biweight midcorrelation between ME and the traits. Lower values in brackets are the P-values for the association test

To identify gene expression profiles associated with fearless and anxiolytic behaviors, we performed a WGCNA to the RNA-seq data [19, 20]. In WGCNA, 11,527 genes (Additional file 4) of the transcriptome dataset are clustered according to co-expression. Average linkage hierarchical clustering was applied to the topological overlap matrix. The branches of highly correlating genes were formed, which were cut and assigned a color. Finally, 12 modules (MEbrown, MEtan, MEblack, MEgreen, MEpurple, MEturquoise, MEred, MEgreenyellow, MEmagenta, MEyellow, MEblue, and MEpink) were identified (Fig. 3a, Additional file 5). The MEgrey module represents unassigned genes. The Module Eigengene (ME) was calculated for each module; it functioned as a representative of the module. The correlations among modules were calculated. Their heatmaps were shown in Fig. 3b. The biweight midcorrelation between each ME and sample trait (binary variables: WT, KO, EPM_WT, and EPM_KO) was calculated based on the bicorAndPvalue() function of WGCNA package in R. Among the 12 modules, the MEgreen module had the most significant correlation with the KO_EPM (Fig. 3c).

### The analysis of MEgreen module

**Fig. 4:**
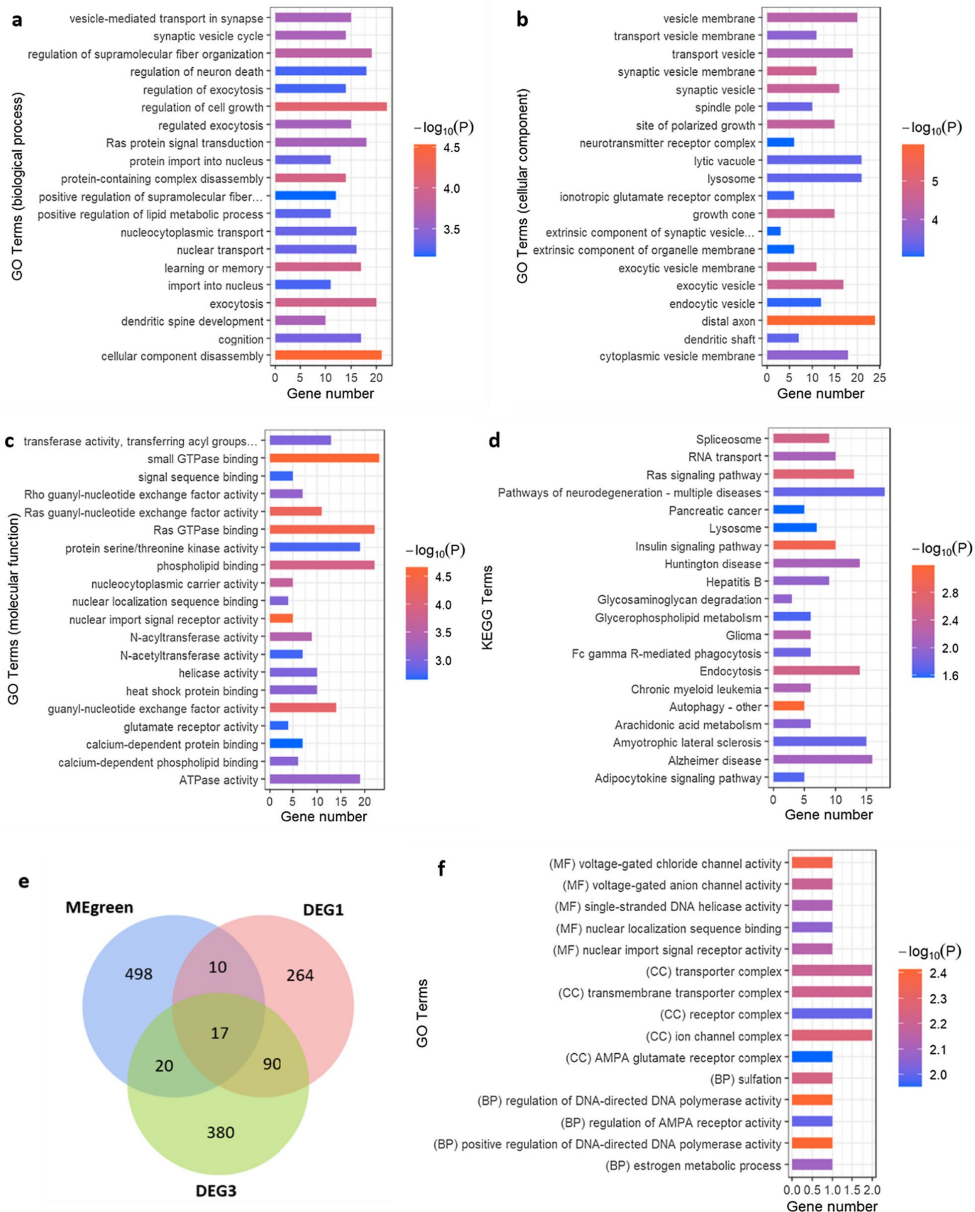
The analysis of MEgreen module genes. **a** The top 20 GO terms relevant to the biological process of MEgreen module. **b** The top 20 GO terms relevant to the cellular component of MEgreen module. **c** The top 20 GO terms relevant to the molecular function of MEgreen module. **d** KEGG enrichment analysis of MEgreen module. The top 20 KEGG terms sorted by the P value. **e** Venn diagram of MEgreen module genes, DEG1 (unshared DEGs in KO_EPM vs. WT_EPM) and DEG3 (unshared DEGs in KO_EPM vs. KO). **f** The top 5 GO terms of the shared genes among MEgreen module, KO_EPM1 and KO_EPM2. GO terms are sorted by the P value. Gene number represents the number of gene types. *BP* biological process, *CC* cellular component, *MF* molecular function

GO enrichment analysis of MEgreen module consisted of three parts, namely, biological process (Fig. 4a), cellular component (Fig. 4b), and molecular function (Fig. 4c). We selected the top 20 GO terms to sort the P value. The potential biological processes revealed by the MEgreen module included cellular component disassembly, regulation of cell growth, protein-containing complex disassembly, learning or memory, and exocytosis. The cellular components included distal axon, synaptic vesicle membrane, exocytic vesicle membrane, growth cone, and exocytic vesicle. The molecular function included small GTPase binding, nuclear import signal receptor activity, Ras GTPase binding, Ras guanyl-nucleotide exchange factor activity, guanyl-nucleotide exchange factor activity, and others*.* GO terms were related to neural regulation include distal axon, synaptic vesicle membrane, exocytic vesicle membrane, nuclear import signal receptor activity, growth cone, and others (Fig. 4a–c, Additional File 6). KEGG enrichment analysis of MEgreen module revealed the higher-level systemic functions that were potentially involved, including autophagy, insulin signaling pathway, Ras signaling pathway, and others (Fig. 4d, Additional File 7).

Venn diagram showed that there were 17 genes which were shared among MEgreen module, DEG1 and DEG3 (Fig. 4e). GO enrichment analysis of the 17 genes showed that they were enriched in the voltage-gated anion channel activity, DNA polymerase activity, transmembrane transporter complex, regulation of AMPA receptor activity and others (Fig. 4f). The Sult1a1, Cnih3, Clcnka and Chtf8 were the main genes of the enrichment.

### Efnb2 knockout on PV+ neurons effects on excitatory post-synaptic potential

**Fig. 5:**
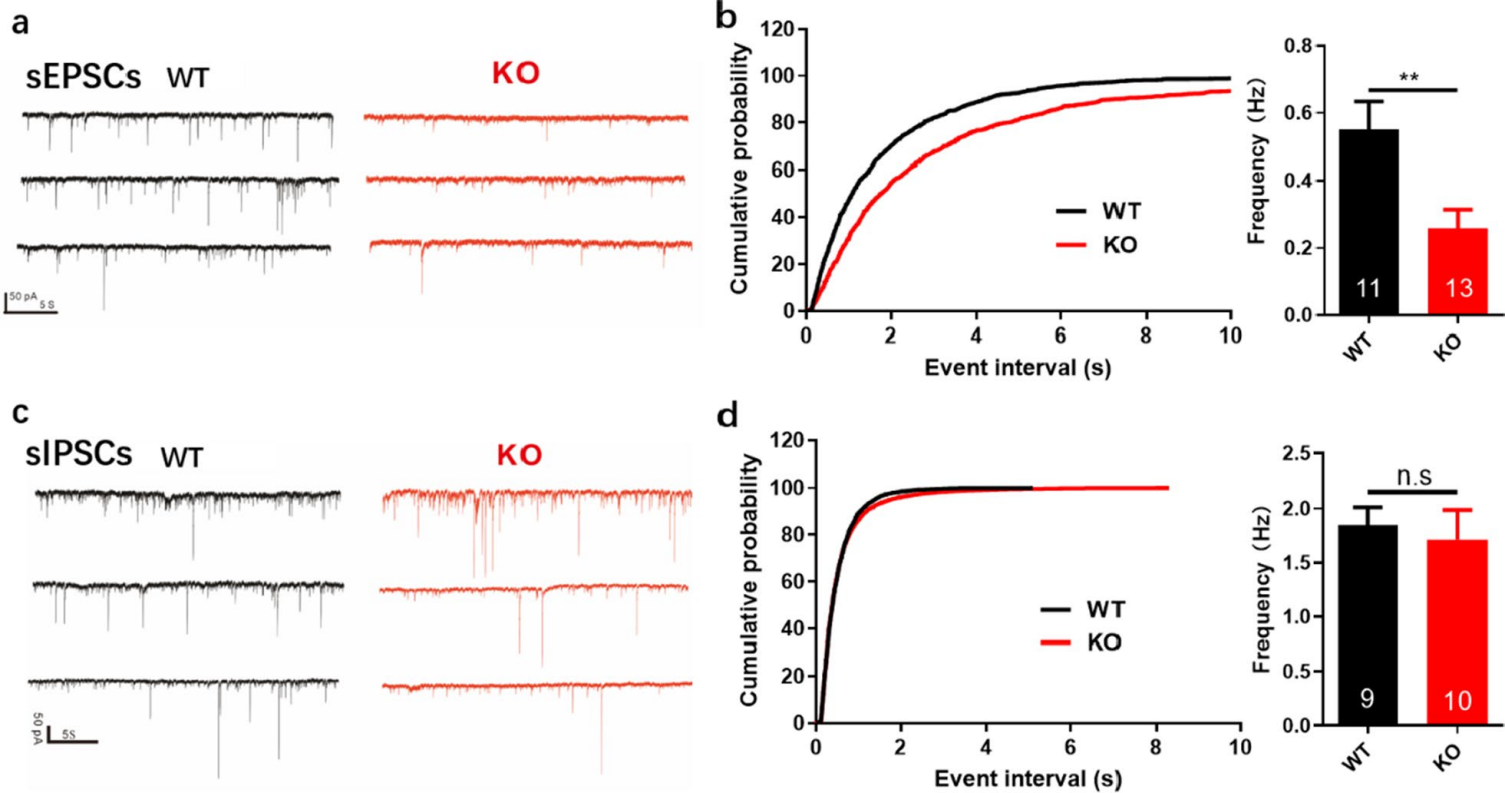
Frequency of spontaneous excitatory post-synaptic currents (sEPSCs) are reduced after Efnb2 knockout. **a** Example traces showing sEPSCs recorded in interneurons of PFC for WT (left) and KO (right). **b** Cumulative probability plot (left) for sEPSCs event interval. Average sEPSCs frequency for the two conditions (right). Data represent mean ± SEM. **c** Example traces showing spontaneous inhibitory post-synaptic currents (sIPSCs) recorded in interneurons of PFC for WT (left) and KO (right). **d** Cumulative probability plot (left) for the event interval of IPSP. Average sIPSCs frequency for the two conditions (right). Data represent mean ± SEM. (male, Unpaired t test, **P ≤ 0.01)

To investigate whether Efnb2 knockout on PV+ neurons of PFC affects the neuronal transmission of pyramidal neurons in medial prefrontal cortex (mPFC), we recorded the spontaneous excitatory post-synaptic currents (sEPSCs) and spontaneous inhibitory post-synaptic currents (sIPSCs) of neurons in the PFC of WT and KO mice. In interneurons from WT mice, the average sEPSCs frequency was 0.5494 ± 0.0846 Hz (n = 11). The average sEPSCs frequency from WT mice was significantly reduced, i.e., 0.2572 ± 0.0566 Hz (n = 13, **P < 0.01; Fig. 5a and b). We investigated the overall incoming inhibitory events by recording sIPSCs of PFC neurons in WT and KO mice, and no difference was found (Fig. 5c and d).

## Discussion

Although a previous study has shown that Efnb2 is expressed in PV+ neurons of PFC [10], its function and relationship with disease progression are unknown. The PV+ neurons of PFC are the primary regulators of emotional behavior [5]. Whether the Efnb2 gene on PV+ neurons can regulate the mental disorder has not been studied. We showed that Efnb2 gene in PV+ neurons of PFC could affect fear and anxiety. The Efnb2 knockout mice exhibited obvious fearless and anxiolytic behaviors.

The PCA results showed a significant difference between KO_EPM and other three groups. In order to further explore the molecular mechanism of fearless behavior, we analyzed the DEGs and identified the genes which were affected by the genotype and the EPM. The DEG1 which were mainly induced by the genotype in KO_EPM mice revealed the molecular function of Toll-like receptor binding. Toll-like receptors (TLRs) have recently emerged as regulators of neuronal survival and developmental neuroplasticity. And the TLR3-deficient mice could be anxious in EPM tasks [21]. The DEG3 which were mainly induced by the EPM in KO_EPM mice revealed the molecular function of actin filament binding. The actin signaling can be affected by the metabotropic glutamate receptors 7 (mGluR7). And the ablation of mGluR7 can lead to the reduced anxiety and depression behaviors related to PFC dysfunction [22]. We found that KO mice induced by EPM will cause the changes in the expression of a series of genes in the mPFC. And these genes were related to the anxiolytic behavior. Therefore, we surmised that knockout of the Efnb2 in PV+ neurons may affect the activity of PV neurons, which further affected the activity of the entire PFC, resulting in the changes in gene expression of KO_EPM.

The WGCNA was used to identify a distinct co-expression network module, which was associated with EPM stress caused by Efnb2 knockout, finding that MEgreen module genes had the most significant correlation with the KO_EPM group. The results of GO enrichment with genes of MEgreen module showed that it is associated with the distal axon, small GTPase binding, and synaptic vesicle membrane. KEGG pathway included Ras signaling and insulin signaling pathways. Previous studies have showed that axon terminals of cholecystokinin-immunoreactive basket cell expresses the type 1 cannabinoid receptor, and the interactions between cholecystokinin and the endogenous cannabinoid system in the basolateral nuclear complex of the amygdala could modulate anxiety-like behavior and fear learning/expression [23]. The Ras plays an essential role in various physiological processes consisting of several enzymes, peptides and receptors [24]. For example, the AT1 receptors could regulate activity of noradrenergic neurons in the brain. And the dysregulation in noradrenaline levels in the brain plays an important role in both anxiety and depression [25]. Insulin signaling accounts for the development of a variety of neuropsychiatric disorders through the interaction between the inflammatory condition and RAS, which impedes brain insulin pathway, resulting in neurobehavioral damage [26].

There were 17 genes which were shared among MEgreen module, DEG1 and DEG3. The main gene enriched by GO was Cnih3 among the 17 genes, which was related to transmembrane transporter complex, ion channel complex and regulation of AMPA receptor activity [27]. Previous studies have shown that AMPA receptors mediate fast excitatory synaptic transmission in the brain [28]. In order to further verify whether the anxiolytic behaviors in KO mice is accompanied by alterations in synaptic excitability, we performed whole-cell voltage clamp recordings in PFC brain region. The average sEPSCs frequencies of WT mice were significantly higher than those of KO mice. The finding is consistent with the results of previous studies. The dmPFC 5-HT6 receptor activation in mice decreased the anxiety. 5-HT6 receptor antagonist SB 271046 could increase sEPSC [29]. This undoubtedly provided synaptic-related evidence for the results of RNA-seq analysis. This study only focused on the changes of the average sEPSCs frequencies between KO and WT mice. The specific regulation mechanism is expected to be studied further in the future.

In conclusion, this article identified a distinct co-expression network module, which has the most significant correlation with KO_EPM by using WGCNA. We characterized the biological processes of this module. The module was related to the distal axon, Ras signaling pathway, and insulin signaling pathway. The whole-cell voltage clamp recordings also showed that Efnb2 gene knock-out could affect the average sEPSCs frequencies. This is the first study to identify the fear- and anxiety-associated genes through WGCNA in Efnb2 knockout mice. We confirmed that Efnb2 on PV+ neurons are important components of fear and anxiety behaviors, which suggested that the identification of the module associated with the fear and anxiety behaviors can provide a treatment strategy and therapeutic target to regulate fear and anxiety symptoms.

## Materials and methods

### Mice

All animal studies were performed in accordance with the National Institute of Health Guide for the Care and Use of Laboratory Animals and were approved by Animal Care and Use Committee in Shanghai Jiao Tong University. Mice were of a mixed CD1/B6 genetic background. All mice have been previously described as having loxP-flanked alleles in Efnb2 [30], which were combined with the parvalbumin (PV)-Cre mice [31].

All mice were housed and had access to food and water in a room with a standard 12 h dark/12 h light cycle. Lights were turned on at 7:00 AM and turned off at 7:00 PM. The room temperature was maintained at 24 ± 1 °C. Behavioral tests were performed between 9:00 AM and 4:00 PM. The behavioral experiments were conducted in the following order: marble burying, cold stress defecation, and EPM. A 3–5 day interval was set between tests. Before all experiments, mice were allowed to habituate in the testing room for 60 min.

### Marble burying

Marble burying test was performed in a cage with the following dimensions: length, 48 cm; width, 35 cm; and height, 20 cm. The cage was filled with 7–8 cm of fresh, autoclaved wood chip bedding (Sharon et al., 2019). Before the test, each mouse was acclimatized to the cage for 10 min and then returned to the home cage. Then, the wood chips were spread evenly. Twenty glass marbles (4 × 5) were placed on top of the wood chips. Subsequently, each mouse was placed carefully back into the corner of cage, as far from marbles as possible. The mouse was allowed to explore for 10 min without being disturbed. Then, the number of marbles buried (50% or more covered) was counted. Before testing the next mouse, the wood chip spread was re-set, and the marbles were cleaned with 75% ethanol.

### Cold stress defecation

Cold stress defecation experiment was performed in a refrigerator at 4 °C. First, the mouse was placed in a cage with a moist paper on the bottom and allowed to acclimate to the environment for 10 min. Then, the cage was placed in the refrigerator at 4 °C for 10 min. The amount of stool defecated during this period was quantified. Before the next mouse was tested, the moist paper in the cage was replaced.

### Elevated plus maze

The EPM apparatus consisted of two open arms and two closed arms of equal dimensions (5 cm width × 35 cm length). Each arm was positioned at a 90° angle relative to the adjacent arms. The closed arms on the opposite sides were enclosed by 15 cm-high walls made of black Plexiglass, and the open arms were open without walls. The four arms were connected by a central area (5 cm width × 5 cm length). The entire EPM was lifted to 30 cm above the floor by the supports. Before the test, the mice were placed in the behavioral room to acclimate for 1 h. At the start of the test, each mouse was placed in the central area facing an open arm and allowed to freely explore the maze for 5 min. Before the next test, the EPM was wiped with a cleaning solution and dried with paper towels. The following variables were scored in the EPM: the percentage of time spent in the open arm (time spent on open arms/time spent in open and closed arms × 100) and the entries into open arms (entries into open arms/entries into open and closed arms × 100).

### Total RNA extraction

The mice were sacrificed by cervical dislocation. Then, the brain was rapidly removed after decapitation. Subsequently, the mPFC region was dissected according to the atlas (Paxinos and Franklin, 4th Edition). The mPFC tissue was collected into the RNase-free tube and kept frozen at − 80 °C until analysis. Finally, 14 mPFC tissue samples were collected, including those from WT mice with loxP-flanked alleles in Efnb2 (WT; n = 4), Efnb2 gene knockout mice (KO; n = 4), WT mice after 1 h of the EPM test (WT_EPM; n = 3), and KO mice after 1 h of the EPM test (KO_EPM; n = 3). Total RNA was isolated and purified using TRIzol reagent (Invitrogen, Carlsbad, CA, USA). RNA amount and purity of each sample were assessed using NanoDrop ND-1000 (NanoDrop, Wilmington, DE, USA). RNA integrity was assessed by Bioanalyzer 2100 (Agilent, CA, USA) with RIN number > 7.0 and confirmed by electrophoresis with denaturing agarose gel.

### RNA sequencing

The construction and sequencing of transcriptome libraries were performed at LC-BIO Bio-tech, Ltd. (Hangzhou, China) according to the protocol recommended by the vendor. Dynabeads Oligo (dT) 25-61005 (Thermo Fisher, CA, USA) was used to purify Poly (A) RNA from 1 μg total RNA. Then, the poly(A) RNA was fragmented into small pieces using Magnesium RNA Fragmentation Module (NEB, cat.e6150, USA) under 94 °C 5–7 min. Then, the cleaved RNA fragments were reverse-transcribed to create the cDNA by SuperScript™ II Reverse Transcriptase (Invitrogen, cat. 1896649, USA), which were next used to synthesize U-labeled second-stranded DNAs with *Escherichia coli* DNA polymerase I (NEB, cat.m0209, USA), RNase H (NEB, cat.m0297, USA) and dUTP Solution (Thermo Fisher, cat.R0133, USA). An A-base was added to the blunt ends of each strand, thereby preparing them for ligation to the indexed adapters. Each adapter contained a T-base overhang for ligating the adapter to the A-tailed fragmented DNA. Single- or dual-index adapters were ligated to the fragments. Size selection was performed with AMPureXP beads. After the heat-labile UDG enzyme (NEB, cat.m0280, USA) treatment of the U-labeled second-stranded DNAs, the ligated products were amplified with PCR. The average insert size for the final cDNA library was 300 ± 50 bp. Finally, we performed the 2 × 150 bp paired-end sequencing (PE150) on an Illumina Novaseq™ 6000 (LC-Bio Technology Co., Ltd., Hangzhou, China).

Cutadapt software (version: cutadapt-1.9) was used to remove the reads that contained adaptor contamination. After removing the low-quality bases and undetermined bases, we used HISAT2 software (version: hisat2-2.0.4) to map reads to the genome. The mapped reads of each sample were assembled using StringTie (version: stringtie-1.3.4d. Linux_x86_64) with default parameters. Then, all transcriptomes from all samples were merged to reconstruct a comprehensive transcriptome using gffcompare software (version: gffcompare-0.9.8. Linux_x86_64). After the final transcriptome was generated, StringTie and ballgown were used to estimate the expression levels of all transcripts and to determine the expression level for mRNAs by calculating FPKM.

### Weighted gene co-expression network analysis (WGCNA)

The WGCNA package was used to perform WGCNA in R studio based on FPKM expression data. The soft threshold (beta) value is equal to 14 which is calculated by the SoftThreshold() function of the WGCNA package. The process of automatic network construction and module detection were completed by the blockwiseModules() function of the WGCNA package. Specifically, Genes were clustered into branches of highly co-expression genes, and modules were identified with the tree cut algorithm.

The trait data contained four sets of binary variables, namely, WT, KO, WT_EPM, and KO_EPM, which were used to calculate the module trait relationships. The groups of interest were set to 1. The others were set to 0. Modules of interest were functionally annotated using *org.Mm.eg.db* annotation data package. The GO enrichment and the KEGG enrichment analysis were preformed using the *clusterProfiler* R package [32].

### Ex vivo whole-cell patch-clamp recording

Whole-cell patch-clamp recordings were performed on WT and KO mice. The mice were first anesthetized by letting them inhale isoflurane and were sacrificed. The brains were rapidly removed and placed in the ice-cooled cutting solution (234 mM sucrose, 3.6 mM KCl, 1.2 mM MgCl_2_, 1.2 mM NaH_2_PO_4_, 12 mM glucose, 2.5 mM CaCl_2_, and 25 mM NaHCO_3_) for 90 s. The 300 µM coronal brain slices of PrL were cut on a vibroslicer (VT 1200S, Leica, Wetzlar, Germany) according to the mouse brain atlas. Then, the slices were immediately transferred into 32 °C artificial cerebrospinal fluid (ACSF, 124 NaCl, 2.5 KCl, 1.25 NaH_2_PO_4_, 1.3 MgSO_4_, 26 NaHCO_3_, 2 CaCl_2_, and 20 d-glucose, equilibrated with 95% O_2_ and 5% CO_2_) for 30 min and at room temperature for 1 h before recordings.

Electrodes (4–6 MΩ) were used for electrophysiological recording of neurons. The sEPSCs were recorded with cs-base interval solution (140 mM Cs-gluconate, 10 mM Hepes, 1.1 mM EGTA, 2 mM MgCl_2_, 3 mM MgATP, and 0.3 mM tris-guanosine triphosphate; pH 7.4 was achieved by adjustment with CsOH) with bath application of picrotoxin (PTX, 100 μM) and clamped at − 70 mV. The sIPSCs were recorded with high-Cl interval solution (140 mM CsCl, 1.1 mM EGTA, 10 mM Hepes, 2 mM MgCl_2_, 3 mM MgATP and 0.3 mM Tris-guanosine triphosphate; pH 7.4 was achieved by adjustment with CsOH) with application of 1 mM kynurenine and clamped at − 70 mV.

Signals were acquired by an Axon 700B amplifier (Axon Instruments, Foster City, CA) and fed into the computer through a Digidata-1550 interface (Axon Instruments) for data analysis (Clampfit 10.7). Access resistance (< 25 MΩ) was considered acceptable throughout all experiments. Moreover, the Signals in which resistance fluctuated in excess of 20% were excluded.

### Statistical analysis

Statistical analyses were performed using Graph Pad Prism software (San Diego, CA, USA). In most cases, the unpaired two-tailed Student’s *t* test was used for normally distributed data. The Mann Whitney test was used for data not normally distributed. The D’Agostino-Pearson test was used for normality test. Data were given as means (± SEM), and differences in means were considered statistically significant at *P* ≤ 0.05. Significance levels [*P* < 0.05 (*), *P* < 0.01(**), *P* < 0.001(***) or *P* < 0.0001(#)] were indicated in the text and figures.

## Supplementary Information

**Additional file 1.** Summary of assembly statistics after Illumina sequencing.

**Additional file 2.** DEGs in KO vs. WT, KO_EPM vs. WT_EPM, KO_EPM vs. KO and WT_EPM, vs. WT.

**Additional file 3.** GO enrichment analysis for genes in DEG1 and DEG2.

**Additional file 4.** Gene list of WGCNA.

**Additional file 5.** List of genes within each WGCNA module.

**Additional file 6.** GO enrichment analysis for genes in MEgreen module.

**Additional file 7.** KEGG enrichment analysis for genes in MEgreen module.

## Data Availability

The data supporting the conclusions of this article are included within its additional files.

## Abbreviations

PFC: Prefrontal cortex
PV+: Parvalbumin-expressing
KO: Knockout
EPM: Elevated plus maze
WGCNA: Weighted gene co-expression network analysis
WT: Wild-type
FPKM: Fragments Per Kilobase of transcript per Million mapped reads
DEG: Differentially expressed genes
ME: Module Eigengene
GO: Gene Ontology
KEGG: Kyoto Encyclopedia of Genes and Genomes
sEPSCs: Spontaneous excitatory post-synaptic currents
sIPSCs: Spontaneous inhibitory post-synaptic currents
mPFC: Medial prefrontal cortex
